# Taking an Alternative Approach to HIV Vaccination

**DOI:** 10.1371/journal.pmed.0030058

**Published:** 2006-01-03

An HIV vaccine still seems to be a long way off, but scientists are pursuing several lines of research that could limit HIV infection. Increasing evidence indicates that the host's natural immunity has a major, albeit usually insufficient, role in limiting HIV-1 infection. Most efforts at stimulating an immune response have been disappointing and have underscored the inability of natural immune responses to control HIV-1 infection in most infected or immunized individuals. Strategies to develop a vaccine include the stimulation of immune responses by manipulating HIV antigens and delivery systems, and by using various adjuvants. One way to achieve an effective immune response to prevent or control HIV infection may be through exploiting the potential of dendritic cells to modulate the immune system. Dendritic cells mediate innate and adaptive immunity against viral infection by providing proinflammatory cytokines and by processing and presenting antigens to T cells.

Now, Xiao-Tong Song, Si-Yi Chen, and colleagues suggest that a molecule that helps regulate dendritic cells can help control not only HIV-specific CD8 ^+^ cytotoxic T lymphocytes (CTLs) and CD4 ^+^ T helper cells but also antibody responses. CD8 ^+^ cytotoxic T cells are the main mediators of viral control, and there is a growing consensus that an effective HIV immunization approach should be capable of inducing vigorous protective CTLs, as well as antibody responses. Song and colleagues suggest that a molecule, the suppressor of cytokine signaling (SOCS) 1, a negative regulator of the Janus kinase/signal transducer and activator-of-transcription (JAK/STAT) pathway in dendritic cells, attenuates cellular signaling in HIV-1 infection. The team has previously noted that SOCS1 helped regulate antigen presentation by dendritic cells, and SOCS1-silenced dendritic cells induced enhanced CTL responses against tumor-associated antigens. [Fig pmed-0030058-g001]

**Figure pmed-0030058-g001:**
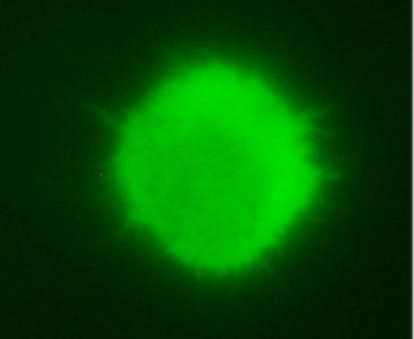
Lentiviral transduced dendritic cells

In a new study, Song and colleagues found that in mice SOCS1-restricted signaling not only controlled the production of proinflammatory cytokines such as IL-12 by dendritic cells but also had a critical role in regulating the anti-HIV immune response. SOCS1-silenced dendritic cells were resistant to HIV envelope-mediated suppression and effectively induced a memory response, with HIV envelope-specific antibody and T cell responses. Furthermore, the potency of the HIV DNA vaccination was significantly enhanced by coimmunization with SOCS1 small interfering RNA (siRNA) expressor DNA. Although the mechanism behind this response is unclear, it may involve the enhanced production of a mixed pattern of Th1- and Th2-polarizing cytokines by SOCS1-silenced dendritic cells.

The findings suggest that a balanced memory humoral and cellular response against HIV could be induced by SOCS1-silenced dendritic cells and SOCS1-siRNA DNA. This SOCS1 silencing strategy could help enhance therapeutic and prophylactic vaccines against HIV and other pathogens. When used with improved HIV immunogens and delivery systems, this vaccination approach may provide a new avenue to enhance weak protective immune responses or to generate broader and stronger responses not only against dominant epitopes but also against weakly immunogenic or cryptic, yet protective, epitopes.

